# Respiratory status of adult patients in the postoperative period of thoracic or upper abdominal surgeries ^1^

**DOI:** 10.1590/1518-8345.2311.2959

**Published:** 2017-12-04

**Authors:** Alana Gomes de Araujo Almeida, Lívia Maia Pascoal, Francisco Dimitre Rodrigo Pereira Santos, Pedro Martins Lima, Simony Fabíola Lopes Nunes, Vanessa Emille Carvalho de Sousa

**Affiliations:** 2 RN; 3 PhD, Adjunct Professor, Centro de Ciências Sociais, Saúde e Tecnologia, Universidade Federal do Maranhão, Imperatriz, MA, Brazil; 4 MSc, Assistent Professor, Universidade do Sul do Maranhão, Imperatriz, MA, Brazil; 5 MSc, Assistent Professor, Centro de Ciências Sociais, Saúde e Tecnologia, Universidade Federal do Maranhão, Imperatriz, MA, Brazil; 6 PhD, Visiting Professor, Instituto de Ciencias da Saúde, Universidade da Integração Internacional da Lusofonia Afro-brasileira, Redenção, CE, Brazil,

**Keywords:** Postanesthesia Nursing, Nursing Assessment, Adult Health, Outcome Assessment (Health Care), Respiratory System, Postoperative Care

## Abstract

**Objective:**

to evaluate the respiratory status of postoperative adult patients by assessing the nursing outcome Respiratory Status.

**Method:**

descriptive, cross-sectional study developed with 312 patients. Eighteen NOC indicators were assessed and rated using a Likert-scale questionnaire and definitions. Descriptive and correlative analysis were conducted.

**Results:**

the most compromised clinical indicators were coughing (65.5%), auscultated breath sounds (55%), and respiratory rate (51.3%). Factors associated with worse NOC ratings in specific clinical indicators were sex, age, pain, and general anesthesia.

**Conclusions:**

certain clinical indicators of respiratory status were more compromised than others in postoperative patients. Patient and context-related variables can affect the level of respiratory compromise.

## Introduction

There is consensus that the quality of nursing care depends greatly on the quality of the nursing assessment, therefore an accurate nursing assessment is crucial to ensure that the planning and implementation of nursing interventions can meet the patient’s needs^1^ ^-^ ^3^. One way of improving the quality of nursing care is implementing standardized nursing languages, including the Nursing Outcomes Classification (NOC). Outcomes are defined as the results of performing a process; in this sense, a positive outcome is the achievement of the goal of the process^4^. 

The NOC contain clinical indicators that are used to evaluate and rate patients’ health status in relation to outcome achievement using five-point Likert-type scales, making this tool an invaluable resource for the nursing assessment. The proper use of the NOC allows the assessment of the current status of the patient and facilitates the identification of changes based on differences in scores documented over time^5^.

In 2015, the American Nurses Association (ANA) released a position statement supporting the use of recognized nursing terminologies to facilitate interoperability of the data collected by nurses. However, many factors have hampered the use of standardized nursing languages in practice such as lack of knowledge about the available nursing languages, lack of integration of standardized nursing languages to the nursing curriculum, lack of professional training on the use of nursing languages, great demand for bureaucratic and administrative services, work overload, and the existence of different terminologies that still lack clear selection criteria or directions for use^6^. In spite of these difficulties, nurses need to understand the importance of standardized nursing documentation and, if necessary, gain the knowledge and skills necessary to effectively include nursing data in standardization efforts^7^. 

Thoracic and upper abdominal surgical procedures often affect the pulmonary volume and capacity, which leads to impaired respiratory function. Patients submitted to those types of surgery are at risk of postoperative respiratory complications such as bronchospasm, atelectasis, infection, and respiratory failure^8^. Approaches to improving quality of care should be aimed at preventing such complications and thus, improving outcomes in the surgical population.

The NOC includes the outcome Respiratory Status, which is defined as “movement of air in and out of the lungs and exchange of carbon dioxide and oxygen at the alveolar level”. The clinical indicators for this outcome are: respiratory rate, respiratory rhythm, depth of inspiration, auscultated breath sounds, airway patency, tidal volume, achievement of expected incentive spirometer, vital capacity, oxygen saturation, pulmonary function tests, accessory muscle use, chest retraction, pursed lip breathing, cyanosis, dyspnea at rest, dyspnea with mild exertion, restlessness, somnolence, diaphoresis, impaired cognition, accumulation of sputum, atelectasis, adventitious breath sounds, impaired expiration, gasping, agonal respirations, grunting, clubbing of fingers, nasal flaring, restlessness, fever, and coughing^5^.

Studies have been developed to test the applicability of NOC outcomes, the validity of NOC indicators, and to translate and adapt NOC outcomes to specific cultural contexts. We found studies assessing NOC outcomes in Intensive Care Unit (ICU) patients^9^, patients with cancer^10^, stroke^11^, diabetes^12^, and congenital heart diseases^13^. However, no study including operational definitions for the nursing outcome Respiratory Status and its applicability to postoperative adult patients was published to this date.

Some advantages of using NOC are the decrease in documentation time allowing more time available for patient care^14^, improved patient satisfaction^15^, reduced variability in the nursing assessment^11^, and improved quality of care by the establishment of parameters for the clinical assessment^16^. However, whenever NOC outcomes are assessed without clear conceptual and operational definitions for clinical indicators, there is uncertainty and subjectivity, which is an important gap in nursing practice. Therefore, this study aimed to evaluate the respiratory status of postoperative adult patients by assessing the nursing outcome Respiratory Status.

## Method

This was a descriptive cross-sectional study. The study was performed between 2014 and 2015 in the post-surgical unit of a large tertiary hospital located in the northeastern Brazil. 

The study involved 312 participants who were hospitalized for surgical treatment of different conditions or diseases. Participants were recruited and assessed during the first 48 hours after surgery, based on the following inclusion criteria: age between 18 and 80 years, and thoracic or upper abdominal surgery. Patients who were using gastric-tube feeding, tracheostomy, or had serious cognitive impairment were excluded because their condition might limit their ability to be examined during the study. 

The sample size was determined by using a formula for infinite populations (n=Zα^2^.P.(1-P)/E^2^) assuming a confidence level (Zα) of 95%, standard error (E) of 5.6%, and a prevalence (P) of respiratory nursing diagnoses at 46.7% according to a previous study^17^. Therefore, the minimum sample size was 305 patients, but 312 patients participated in the study.

Data collection was performed by a research team composed by undergraduate nursing students led by the main researcher. Before data collection, the team participated in a training about the specific steps of the physical examination of the chest and lungs and took a written test that determined their ability to perform the physical examination. Data collection was conducted individually with each patient, after consent, at a single time point during hospitalization. Each patient was interviewed in the hospital ward and received a guided physical examination based in an instrument specially developed for the study.

Interviews and physical examinations aimed to collect data for the assessment of 18 NOC indicators belonging to the Respiratory Status outcome. The instrument also included demographic variables such as gender, age, marital status, and education, and clinical information such as surgical procedure and indication for surgery.

Following data collection, the researchers met to discuss and rate the level of compromising of each of the 18 clinical indicators by consensus, using the NOC ratings. Conceptual and operational definitions that were obtained and adapted from the literature^18^ ^-^ ^19^ were used as a basis to rate each indicator into five levels: 1 representing the highest degree of severity and 5 representing the lowest level of severity or lack of compromise. Indicators were considered compromised when they were rated below or equal to 4. 

The raw data were stored in Microsoft Excel format and statistical analyses were performed using the Statistical Package for the Social Sciences (SPSS) version 21.0. For the descriptive analysis, absolute mean values and ranges of the variables of interested are reported. To check the normality assumption, the Kolmogorov-Smirnov test was applied. 

Odds Ratio with confidence intervals, Qui-square, and Fisher tests were applied in the analysis of associations between the clinical indicators and gender, presence of pain, and type of anesthesia. The Mann Whitney test was used to compare groups on age and presence of compromised clinical indicators. The tests were applied at significance level equal to 0.05. 

The study protocol was approved by the Ethics Committee of the Federal University of Maranhão, according to Protocol no. 629.315. Informed written consent was obtained from all patients before enrollment in compliance with the Resolution n^o^. 466/12 about the Guidelines and Regulatory Standards for research involving human beings of the National Health Council.

## Results

Of the 312 participants (mean age 38, SD 16, range 18-78), 67.9% are male, 46.8% never were married or lived in a paired relationship, and 54% have had primary or secondary education. Table 1 show the clinical characteristics of the patients. Most patients (56.5%) had never smoked. The most frequent surgery performed was exploratory laparotomy (78.2%) and 70.6% patients underwent local or regional anesthesia. On examination, all patients had normal vital parameters. 

**Table 1:** Distribution of patients according to smoking, type of surgery, type of anesthesia, complaints, and vital parameters. Imperatriz, MA, Brazil, 2014-2015

**Variables**		N*			**%**	
**Smoking**						
	**Never smoker**	**175**			**56.5**	
	**Passive smoker**	**18**			**5.8**	
	**Quitter smoker**	**42**			**13.5**	
	**Current smoker**	**75**			**24.2**	
**Type of Surgery**						
	**Exploratory laparotomy**	**244**			**78.2**	
	**Thoracotomy**	**66**			**21.1**	
	**Cholecystectomy**	**46**			**14.7**	
	**Appendectomy**	**32**			**10.3**	
	**Other**	**50**			**16.9**	
**Type of anesthesia**						
	**Local or regional anesthesia**	**204**			**70.6**	
	**General anesthesia**	**85**			**29.4**	
**Complaints**						
	**Pain**	**177**			**57**	
	**Cough**	**105**			**33.6**	
**Vital parameters**		**N** ^**†**^	**Min**	**Max**	**Mean**	**SD**
	**Respiratory rate**	**312**	**11**	**50**	**21.29**	**4.45**
	**Heart rate**	**312**	**42**	**132**	**80.40**	**16.32**
	**SpO**_**2**_	**309**	**61**	**99**	**93.91**	**3.97**

*N - Some patients were submitted to more than 1 type of surgery, †N - Variations in N ocurred because not all questions were answered by all subjects.

Findings from the assessment of the NOC outcome Respiratory Status are summarized on Table 2, from the most to the less compromised clinical indicator. Coughing, auscultated breath sounds, and respiratory rate were the most compromised clinical indicators, as each of them were rated by the evaluators at some level of compromise (1 to 4) on more than 50% of the assessments.

**Table 2:** Distribution of Nursing Outcomes Classification (NOC) indicators of Respiratory Status by level of severity. Imperatriz, MA, Brazil, 2014-2015

NOC Indicators*	**Severe**			**Substantial**			**Moderate**			**Mild**			**None**	
	**n**	**%**		**n**	**%**		**n**	**%**		**n**	**%**		**n**	**%**
**Coughing**	**7**	**6.4**		**8**	**7.3**		**8**	**7.3**		**49**	**44.5**		**38**	**34.5**
**Auscultated breath sounds**	**34**	**10.9**		**50**	**16.1**		**33**	**10.6**		**54**	**17.4**		**140**	**45.0**
**Respiratory rate**	**8**	**2.6**		**6**	**1.9**		**30**	**9.6**		**116**	**37.2**		**152**	**48.7**
**Oxygen saturation**	**3**	**1.0**		**5**	**1.6**		**26**	**8.4**		**110**	**35.6**		**165**	**53.4**
**Accessory muscle use**	**8**	**2.6**		**9**	**2.9**		**36**	**11.6**		**84**	**27.0**		**174**	**55.9**
**Depth of inspiration**	**5**	**1.6**		**--**	**--**		**8**	**2.6**		**78**	**25.2**		**218**	**70.6**
**Adventitious breath sounds**	**4**	**1.3**		**4**	**1.3**		**12**	**3.9**		**28**	**9.0**		**262**	**84.5**
**Respiratory rhythm**	**2**	**0.6**		**6**	**1.9**		**5**	**1.6**		**28**	**9.1**		**268**	**86.7**
**Chest retraction**	**--**	**--**		**5**	**1.6**		**10**	**3.2**		**23**	**7.4**		**273**	**87.8**
**Dyspnea with mild exertion**	**17**	**5.4**		**--**	**--**		**17**	**5.4**		**1**	**0.3**		**277**	**88.8**
**Accumulation of sputum**	**4**	**1.3**		**4**	**1.3**		**7**	**2.3**		**13**	**4.2**		**282**	**91.0**
**Dyspnea at rest**	**5**	**1.6**		**4**	**1.3**		**2**	**0.6**		**4**	**1.0**		**298**	**95.5**
**Diaphoresis**	**--**	**--**		**4**	**1.3**		**6**	**1.9**		**3**	**1**		**296**	**95.8**
**Somnolence**	**2**	**0.6**		**5**	**1.6**		**2**	**0.6**		**4**	**1.9**		**293**	**95.1**
**Pursed lips breathing**	**2**	**0.6**		**--**	**--**		**--**	**--**		**5**	**1.6**		**303**	**97.7**
**Cyanosis**	**--**	**--**		**5**	**1.6**		**--**	**--**		**1**	**0.3**		**304**	**98.1**
**Restlessness**	**--**	**--**		**--**	**--**		**--**	**--**		**3**	**1.0**		**307**	**99.0**
**Nasal flaring**	**--**	**--**		**--**	**--**		**1**	**0.3**		**2**	**0.6**		**307**	**99.0**

*NOC - Nursing Outcomes Classification.

To test the correlations between impairment of clinical indicators and patients’ personal and clinical characteristics, we studied the bivariate effect of different variables on the NOC ratings (Tables 3 and 4). 

**Table 3:** Correlations between Nursing Outcomes Classification (NOC). indicators and personal characteristics of the patients. Imperatriz, MA, Brazil, 2014-2015

**Variables**	**Present**	**Absent**	**Statistics**
**Age**	**Mean ranks**		
**Auscultated breath sounds**	**140.95**	**171.96**	*p* =0.002*
**Oxygen saturation**	**171.50**	**138.74**	*p* =0.001*
**Gender**	**Male**	**Female**	
**Auscultated breath sounds**			*p* **=0.015** ^**†**^
**Compromised**	**126**	**45**	**OR=0.55**
**Non-compromised**	**85**	**55**	**IC95%=0.341-0.892**
**Depth of inspiration**			*p* **=0.007** ^**†**^
**Compromised**	**72**	**19**	**OR=0.45**
**Non-compromised**	**138**	**80**	**IC95%=0.256-0.810**
**Adventitious breath sounds**			*p* **=0.029** ^**†**^
**Compromised**	**39**	**9**	**OR=0.43**
**Non-compromised**	**171**	**91**	**IC95%=0.201-0.935**

*P-value based on the exact Mann-Whitney test. †*P-value* based on the Pearson’s chi-square test.

**Table 4:** Correlations between Nursing Outcomes Classification (NOC). indicators and clinical characteristics of the patients. Imperatriz, MA, Brazil, 2014-2015

**Variables**		**Present**	**Absent**	**Statistics**
**Pain**				
**Dyspnea with mild exertion**				*p* =0.003*
	**Compromised**	**28**	**7**	**OR=3.40**
	**Non-compromised**	**149**	**127**	**CI95%=1.441-8.068**
**Auscultated breath sounds**				*p* =0.029*
	**Compromised**	**106**	**64**	**OR=1.65**
	**Non-compromised**	**70**	**70**	**CI95%=1.052-2.608**
**Cyanosis**				*p* **=0.039** ^**†**^
	**Compromised**	**6**	**0**	**OR=0.56**
	**Non-compromised**	**170**	**133**	**CI95%=0.508-0.620**
**Respiratory rhythm**				*p* =0.026*
	**Compromised**	**17**	**24**	**OR=0.47**
	**Non-compromised**	**160**	**107**	**CI95%=0.243-0.924**
**Type of Anesthesia**		**General**	**Local/Regional**	
**Somnolence**				*p* =0.003*
	**Compromised**	**9**	**4**	**OR=5.91**
	**Non-compromised**	**75**	**197**	**CI95%=1.767-19.768**
**Retracción torácica**				*p* =0.000*
	**Compromised**	**21**	**14**	**OR=4.43**
	**Non-compromised**	**64**	**189**	**CI95%=2.128-9.222**
**Dyspnea with mild exertion**				*p* =0.000*
	**Compromised**	**18**	**14**	**OR=3.64**
	**Non-compromised**	**190**	**67**	**CI95%=1.719-7.733**
**Diaphoresis**				*p* **=0.047** ^**†**^
	**Compromised**	**7**	**5**	**OR=3.51**
	**Non-compromised**	**78**	**196**	**CI95%=1.084-11.417**
**Accessory muscle use**				*p* =0.022*
	**Compromised**	**46**	**80**	**OR=1.81**
	**Non-compromised**	**39**	**123**	**CI95%=1.088-3.023**
**Cyanosis**				*p* **=0.002** ^**†**^
	**Compromised**	**5**	**0**	**OR=0.28**
	**Non-compromised**	**202**	**80**	**CI95%=0.236-0.341**
**Respiratory rate**				*p* =0.024*
	**Compromised**	**33**	**110**	**OR=0.55**
	**Non-compromised**	**51**	**94**	**CI95%=0.330-0.927**

*P-value based on the Pearson’s chi-square test. †*P-value* based on the Fisher’s exact test.

The table 3 data shows that older patients had worse NOC ratings to oxygen saturation than younger patients (Mann-Whitney: mean rank 172 vs. 139; *P* = 0.001), but younger patients had worse ratings to auscultated breath sounds than the older ones (mean rank 172 vs. 141; *P* = 0.002). Male patients had less chance of having compromised auscultated breath sounds (odd ratio: 0.55, 95% CI: 0.34-0.89), depth of inspiration (odd ratio: 0.45, 95% CI: 0.26-0.81), and adventitious breath sounds (odd ratio: 0.43, 95% CI: 0.20-0.94) compared to females. 

As pointed out in table 4 patients with pain had more chances of having dyspnea with mild exertion (odd ratio: 3.40, 95% CI: 1.44-8.07) and altered auscultated breath sounds (odd ratio: 1.65, 95% CI: 1.05-2.61) compared to those without pain. Patients without pain also had less chances of having compromised respiratory rhythm (odd ratio: 0.47, 95% CI: 0.24-0.92). Surprisingly, patients with pain had less chances of having cyanosis (odd ratio: 0.56, 95% CI: 0.51-0.62). 

General anesthesia was related with an increased chance of having compromised somnolence (odd ratio: 5.91, 95% CI: 1.77-19.77), chest retraction (odd ratio: 4.43, 95% CI: 2.13-9.22), dyspnea with mild exertion (odd ratio: 3.64, 95% CI: 1.71-7.73), diaphoresis (odd ratio: 3.51, 95% CI: 1.08-11.41), and accessory muscle use (odd ratio: 1.81, 95% CI: 1.08-3.02). On the other hand, patients submitted to general anesthesia had less chance of having cyanosis (odd ratio: 0.28, 95% CI: 0.24-0.34). Patients who received local/regional anesthesia also had less chances of having compromised respiratory rate (odd ratio: 0.55, 95% CI: 0.33-0.93).

## Discussion

This study assessed 312 adult patients in the postoperative period, aiming to verify the level of compromise of 18 clinical indicators of the NOC outcome Respiratory Status. Coughing, auscultated breath sounds, and respiratory rate were the most compromised clinical indicators.

The postoperative period of large surgeries on the thoracic and upper abdominal areas is usually accompanied by impairments on the pulmonary mechanics. During normal respiration, the diaphragm produces a negative pressure inside the thorax that pulls air into the lungs. Surgical incision affects the integrity of the respiratory muscles and interfere in local nerve impulses that are involved in the mechanics of respiration^20^. Large surgeries are also associated with pain, respiratory muscle dysfunction, and reduced lung airflow. All these changes trigger an increase or decrease in ventilation and alter the frequency and depth of respiration because of the stress caused by the surgery^21^. These factors combined with the anesthesia effects explain the presence of the responses that we detected in the study.

The bivariate analysis showed that age was significantly related with compromised oxygen saturation (in favor of younger patients) and compromised auscultated breath sounds (in favor of older patients). Aging is a well-known risk factor for postoperative pulmonary complications^22^. Aged patients are more prone to develop desaturation than younger patients because of their reduced physiologic reserve. Besides that, all general anesthetics produce cardiovascular depression that can be augmented in the aged patient^23^.

Controversially, the younger patients were more compromised than the older ones in relation to auscultated breath sounds. A possible explanation to this finding is the fact that most of these patients were under surgical treatment following violent injuries, such as perforations caused by gunshot or knife wound. Penetrating chest trauma opens the pleural space to the atmosphere leading to pulmonary complications such as collapsed lung and pneumothorax, and the effects of these complications can persist on the postoperative period^24^.

Female patients were more prone than male patients to present compromise of three clinical indicators. The literature shows that there are differences by gender on the pulmonary function of adults. Women generally have reduced airway diameter, lower resting lung diffusing capacity, smaller lung volumes, and lower maximal expiratory flow rates than men, even when corrected for age and standing height^25^. Consequently, women are more likely to experience greater mechanical limits to expiratory flow compared with men, creating a smaller maximal flow^26^, which can possibly explain our results.

Pain was also significantly related with respiratory compromise. Post-surgical pain is one of the most common symptoms on the postoperative period (experienced by 57% of our study), and can be an important determinant for the patients’ recovery. Pain is an acute nociceptive response caused by thermal, mechanical, or chemical injury, which activate free nerve endings. In response to postoperative pain, patients tend to alter their respiratory pattern resulting in reduced inspiratory depth and compromised respiratory function^27^. We have no explanation to the finding that pain acted as a protection factor for the occurrence of cyanosis. Maybe the presence of pain made nurses more attentive to these patients’ needs, but additional observations are needed. 

General anesthesia was related with compromise in several clinical indicators, while local/regional anesthesia was a protection-factor. One of the causes of respiratory complications is the residual effect of anesthetic drugs on the body. Opioid agents used in general anesthesia produce a shift to the right of the carbon dioxide curve, while inhalational agents alter the pulmonary vasoconstriction, leading to respiratory depression and impairment of the pulmonary function^28^. In addition, general anesthesia reduces lung volume and capacity, reduces chest and lung compliance, and affects the diaphragm movement. On the other hand, local and regional anesthesia do not affect the respiratory function, hence, patients who receive local anesthesia generally do not have serious postoperative complications^29^. We have no explanation to the finding that general anesthesia acted as a protection factor for the occurrence of cyanosis, but the literature shows that postoperative cyanosis is usually more related to a patient’s disease or baseline condition than type of anesthesia^30^.

Some limitations of this study must be considered. Since data was collected by different people, differences in the clinical assessment of respiratory changes may have occurred despite training. In addition, the fact that inclusion was restricted to patients on the first 48 hours after surgery may be interpreted as a limitation since some patients could not answer some questions because of their altered health status, and their relatives had to provide some information. Despite that, the study objectives were achieved and the findings can be useful to promote nursing awareness of common respiratory alterations in postoperative patients. 

## Conclusion

The certain clinical indicators of compromised Respiratory Status are more prevalent than others in postoperative patients, and that age, gender, pain, and general anesthesia can affect the chance of having a more severe level of compromise. The systematic approach used for evaluating the respiratory status of the patients can help nurses to early detect respiratory impairment, which can be life-threatening without prompt recognition and assistance.
